# Update: Severe Respiratory Illness Associated with Middle East Respiratory Syndrome Coronavirus (MERS-CoV) — Worldwide, 2012–2013

**Published:** 2013-06-14

**Authors:** Div of Global Migration and Quarantine, Div of Healthcare Quality Promotion, National Center for Emerging and Zoonotic Infectious Diseases, Office for Emergency Preparedness and Response, National Institute of Occupational Safety and Health, Div of Global Health Protection (proposed), Center for Global Health; Div of Viral Diseases, National Center for Immunization and Respiratory Diseases, Paul A. Gastañaduy

**Affiliations:** EIS Officer, CDC

CDC continues to work in consultation with the World Health Organization (WHO) and other partners to better understand the public health risk posed by the Middle East Respiratory Syndrome Coronavirus (MERS-CoV), formerly known as novel coronavirus, which was first reported to cause human infection in September 2012 (1–4). The continued reporting of new cases indicates that there is an ongoing risk for transmission to humans in the area of the Arabian Peninsula. New reports of cases outside the region raise concerns about importation to other geographic areas. Nosocomial outbreaks with transmission to health-care personnel highlight the importance of infection control procedures. Recent data suggest that mild respiratory illness might be part of the clinical spectrum of MERS-CoV infection, and presentations might not initially include respiratory symptoms. In addition, patients with comorbidities or immunosuppression might be at increased risk for infection, severe disease, or both. Importantly, the incubation period might be longer than previously estimated. Finally, lower respiratory tract specimens (e.g., sputum, bronchoalveolar lavage, bronchial wash, or tracheal aspirate) should be collected in addition to nasopharyngeal sampling for evaluation of patients under investigation. An Emergency Use Authorization (EUA) was recently issued by the Food and Drug Administration (FDA) to allow for expanded availability of diagnostic testing in the United States.

As of June 7, 2013, a total of 55 laboratory-confirmed cases have been reported to WHO. Illness onsets have occurred during April 2012 through May 29, 2013 (Figure 1). All reported cases were directly or indirectly linked to one of four countries: Saudi Arabia, Qatar, Jordan, and the United Arab Emirates (Figure 2). Most cases (40) were reported by Saudi Arabia. Four countries, the United Kingdom (UK), Italy, France, and Tunisia, have reported cases in returning travelers and their close contacts (5–8). Ill patients from Qatar and the United Arab Emirates have been transferred to hospitals in the UK and Germany. To date, no cases have been reported in the United States. WHO and CDC have not issued any travel advisories at this time; updated information for travelers to the Arabian Peninsula is available at http://wwwnc.cdc.gov/travel/notices/watch/coronavirus-arabian-peninsula.

The median age of patients is 56 years (range: 2–94 years), with a male-to-female ratio of 2.6 to 1.0. All patients were aged ≥24 years, except for two children, one aged 2 years and one aged 14 years. All patients had respiratory symptoms during their illness, with the majority experiencing severe acute respiratory disease requiring hospitalization. Thirty-one of the 55 patients are reported to have died (case-fatality rate: 56%) (5–8). Two cases in Tunisia, in siblings whose father’s illness was a probable case, and a case from the UK, were in persons with mild respiratory illnesses who were not hospitalized (5,9). Information was not available for all cases; however, several patients had accompanying gastrointestinal symptoms, including abdominal pain and diarrhea, and many cases occurred among persons with chronic underlying medical conditions or immunosuppression, as reported to WHO (5,9).

The original source(s), route(s) of transmission to humans, and the mode(s) of human-to-human transmission have not been determined. Eight clusters (42 cases) have been reported by six countries (France, Italy, Jordan, Saudi Arabia, Tunisia, and the UK) (5) among close contacts or in health-care settings and provide clear evidence of human-to-human transmission of MERS-CoV. The first documented patient-to-patient nosocomial transmission in Europe was confirmed recently in France (10). The first French patient, a man aged 64 years with a history of renal transplantation, became ill on April 22, 2013, within 1 week after returning from Dubai. He presented with fever and diarrhea. Pneumonia was diagnosed incidentally on radiographic imaging, and he subsequently died with severe respiratory disease. The secondary case is in a man aged 51 years on long-term corticosteroids who shared a room with the index patient during April 26–29 and who remains hospitalized on life support. The incubation period for the secondary case was estimated to be 9–12 days; this is longer than the previously estimated 1–9 days (10). A larger cluster, consisting of 25 cases including 14 deaths, ongoing since April 2013 in the region of Al-Ahsa in eastern Saudi Arabia, also has included cases linked to a health-care facility (5). Cases have included health-care personnel and family contacts. An additional five cases, not linked to the cluster in Al-Ahsa, were reported recently in another region of eastern Saudi Arabia (5). Thus far, no evidence of sustained community transmission beyond the clusters has been reported in any country.

In some instances, sampling with nasopharyngeal swabs did not detect MERS-CoV by polymerase chain reaction (PCR); however, MERS-CoV was detected by PCR in lower respiratory tract specimens from these same patients. In the two patients reported by France, nasopharyngeal specimens were weakly positive or inconclusive, whereas bronchoalveolar lavage and induced sputum were positive (10).

## CDC Guidance

In consultation with WHO, the period for considering evaluation for MERS-CoV infection in persons who develop severe acute lower respiratory illness days after traveling from the Arabian Peninsula or neighboring countries* has been extended from within 10 days to within 14 days of travel. Persons who develop severe acute lower respiratory illness within 14 days after traveling from the Arabian Peninsula or neighboring countries should be evaluated according to current guidelines (available at http://www.cdc.gov/coronavirus/mers/case-def.html). Persons whose respiratory illness remains unexplained and who meet criteria for “patient under investigation” should be reported immediately to CDC through state and local health departments. Persons who develop severe acute lower respiratory illness who are close contacts† of a symptomatic traveler who developed fever and acute respiratory illness within 14 days of traveling from the Arabian Peninsula or neighboring countries may be considered for evaluation for MERS-CoV. In addition, CDC recommends that clusters of severe acute respiratory illness be investigated and, if no obvious etiology is identified, local public health officials be notified and testing for MERS-CoV conducted, if indicated.

To increase the likelihood of detecting MERS-CoV, CDC recommends collection of specimens from different sites (e.g., a nasopharyngeal swab and a lower respiratory tract specimen, such as sputum, bronchoalveolar lavage, bronchial wash, or tracheal aspirate). Specimens should be collected at different times after symptom onset, if possible. Lower respiratory tract specimens should be a priority for collection and PCR testing; stool specimens also may be collected. Specimens should be collected with appropriate infection control precautions (available at http://www.cdc.gov/coronavirus/mers/case-def.html).

Testing of specimens for MERS-CoV currently is being conducted at CDC. FDA issued an EUA on June 5, 2013, to authorize use of CDC’s novel coronavirus 2012 real-time reverse transcription–PCR assay (NCV-2-12 rRT-PCR assay) to test for MERS-CoV in clinical respiratory, blood, and stool specimens. This EUA is needed because, at this time, there are no FDA-approved tests that identify MERS-CoV in clinical specimens. This assay will be deployed to Laboratory Response Network (LRN) laboratories in all 50 states over the coming weeks. Updated information about laboratories with the capacity to conduct MERS testing with the NCV-2-12 rRT-PCR assay will be provided on CDC’s MERS website (http://www.cdc.gov/coronavirus/mers/case-def.html).

In consultation with WHO, the definition of a probable case of MERS-CoV infection has been updated to also include persons with severe acute respiratory illness with no known etiology with an epidemiologic link to a confirmed case of MERS-CoV infection. Until the transmission characteristics of MERS-CoV are better understood, patients under investigation and probable and confirmed cases should be managed in health-care facilities using standard, contact, and airborne precautions. As information becomes available, these recommendations will be reevaluated and updated as needed.

Recommendations and guidance on case definitions, infection control (including use of personal protective equipment), case investigation, and specimen collection and testing, are available at the CDC MERS website (http://www.cdc.gov/coronavirus/mers/index.html). The MERS website contains the most current information and guidance, which is subject to change. State and local health departments with questions should contact the CDC Emergency Operations Center (770-488-7100).

## Figures and Tables

**FIGURE 1:**
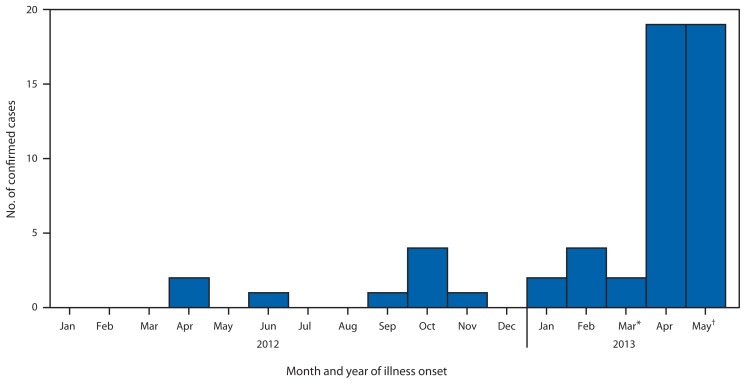
Number of confirmed cases of Middle East Respiratory Syndrome Coronavirus (MERS-CoV) (N = 55) reported as of June 7, 2013, to the World Health Organization, by month of illness onset — worldwide, 2012–2013 ^*^ Case count for March assumes that the two cases included in the March 23, 2013 WHO announcement had symptom onset during March 2013. ^†^Case count for May 2013 assumes that six recently reported cases had symptom onset during May 2013.

**FIGURE 2:**
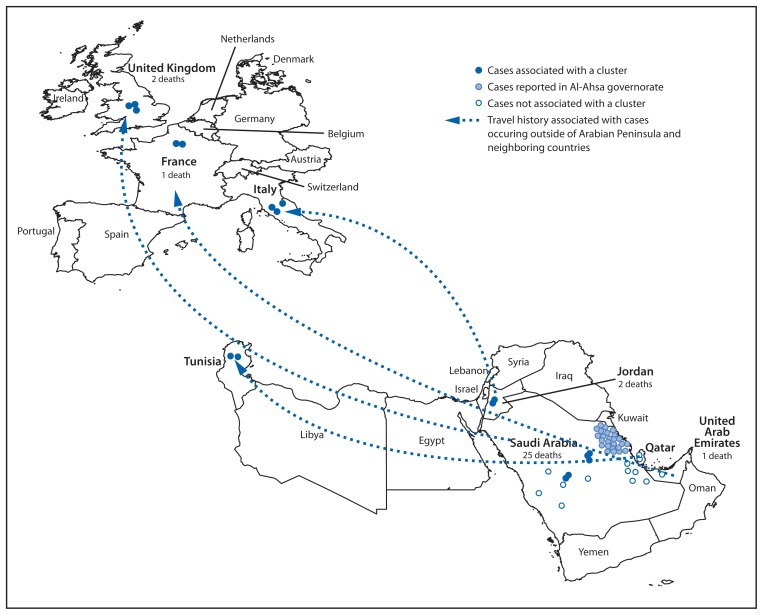
Confirmed cases^*^ of Middle East Respiratory Syndrome Coronavirus (MERS-CoV) (N =55) reported as of June 7, 2013, to the World Health Organization, and history of travel from the Arabian Peninsula or neighboring countries within 14 days of illness onset — worldwide, 2012–2013 ^*^ Dots representing the cases are not geographically representative of the exact location of the residence of the patient.

## Footnotes

* Countries considered to be on or neighboring the Arabian Peninsula include Bahrain, Iraq, Iran, Israel, Jordan, Kuwait, Lebanon, Oman, Palestinian Territories, Qatar, Saudi Arabia, Syria, the United Arab Emirates, and Yemen.

† Close contacts are defined as 1) persons who provided care for the patient, including health-care personnel and family members, or who had other similarly close physical contact, or 2) persons who stayed at the same place (e.g., lived with or visited) as the patient while the patient was ill.
